# Filial Imprinting: Behaviour and Neurobiology

**DOI:** 10.3390/bs16050741

**Published:** 2026-05-09

**Authors:** Brian J. McCabe

**Affiliations:** 1 Department of Zoology, University of Cambridge, Downing Street, Cambridge CB2 3EJ, UK; bjm1@cam.ac.uk; 2 Robinson College, University of Cambridge, Grange Road, Cambridge CB3 9AN, UK

**Keywords:** visual imprinting, domestic chick, learning, memory, IMM, IMHV, early learning, behavioural attachment, intermedial medial mesopallium, sleep

## Abstract

Research on filial imprinting has yielded insights into a range of behavioural and neurobiological phenomena, and these insights have in turn fed back to elucidate behavioural development. This review will summarize important stages in this progression, with emphasis on the neural mechanisms underlying visual filial imprinting in the domestic chick. Imprinting entails recognition of stimuli, in terms of both form and certain abstract features. A striking property of imprinting is the development of a preference for a stimulus slightly different from one that has become familiar, a property having profound implications for survival. Compelling evidence indicates that the intermediate and medial mesopallium (IMM) in the chick forebrain is a site of memory encoding for imprinting. In addition, processes within the IMM are intimately associated with learning capacity in the absence of specific experience (a predisposition). Electrophysiological, neuroanatomical, pharmacological, biochemical and ablation studies have implicated the IMM in the recognition of individual conspecifics, and recent research has elucidated the underlying neurobiological mechanisms at the cellular and sub-cellular levels. Results from studies of imprinting in chicks have led to the discovery of analogous processes in humans and promise to yield insights into cognitive development in both species.

## 1. Introduction

It has been known since antiquity that the young of certain vertebrate species will follow adult conspecifics, usually their parents, very soon after birth or hatching. This is readily observed in precocial species where infants, being relatively mobile, can avoid the hazards of isolation by staying close to protective companions. Precocial animals have provided a wealth of experimental data about early social attachment owing to the relative ease with which behavioural observations can be made. Given that postnatal isolation can be very dangerous irrespective of locomotor capability, it would not be surprising if the young of at least some altricial species exhibit a similar tendency, and indeed this idea has framed thinking about human filial behaviour (Robledo et al., 2022). It is therefore reasonable to suppose that the study of early social attachment in precocial species is likely to lead to insights into the neurobiology of social attachment and behavioural development in others.

It takes only a little observation to appreciate that as well as showing following behaviour, young animals cleaving to their parents learn the latter’s characteristics, demonstrated by the infant’s ability to discriminate familiar from novel individuals. This is the phenomenon of filial imprinting (referred to here simply as ‘imprinting’), which can involve several sensory modalities. Olfactory imprinting has been demonstrated in the guinea pig *Cavia porcellus* (Carter & Marr, 1970) and the spiny mouse *Acomys cahirinus* (Porter & Etscorn, 1974), taking advantage of these species’ reliance on the sense of smell. For example, exposure of neonatal spiny mice to an unfamiliar odour (either cinnamon or cumin) gives rise to a preference for that odour. This process has a sensitive period, and exposure to the odour within this period can generate a preference lasting up to 10 days (Janus, 1993).

Imprinting in some species is readily studied with visual stimuli. Precocial birds such as domestic chickens and ducks show visual imprinting to a remarkable extent. This process is rapid and, with adequate exposure to an imprinting stimulus, can influence behaviour at least until the onset of adulthood (Bolhuis et al., 1989). Imprinting in domestic chicks is readily studied in the laboratory, and the many advantages of doing so has led to a large accumulation of data and many insights into the properties of this form of learning (Bateson, 1966; Bolhuis, 1991; McCabe, 2019; Sluckin, 1972). Despite the ethical and practical problems raised by studying human neonates, an analogous process has been described in our own species. It is appropriate to discuss this in a later section in view of the influence of the work on precocial birds, which will be described first.

Imprinting in chicks and ducklings occurs during a sensitive period a few days after hatching, described graphically by Spalding (1873). The sensitive period is pharmacologically manipulable (Parsons & Rogers, 2000, 1997) and has been shown by Yamaguchi et al. (2012) to critically involve thyroid hormones. There are other determinants of the sensitive period, however, since exogenous thyroid hormones cannot extend the sensitive period in domestic chicks beyond approximately 10 days after hatching (Yamaguchi et al., 2012).

I describe here a program of research on imprinting in the domestic chick, conducted at the behavioural and neurobiological levels. I will then discuss the relevance of some of these findings to human studies.

## 2. Learning the Features of an Imprinting Stimulus

In general, and within temporal limits, the longer a domestic chick is exposed to an imprinting stimulus, the more marked is the chick’s preference for the stimulus, relative to novel stimuli (Bateson & Jaeckel, 1976; Bolhuis et al., 2000). During the first few days after hatching, there is evidence that imprinting becomes more stable and is susceptible to interaction with predispositions in the early phases of the process (Lemaire et al., 2021).

Visual imprinting is thus not instantaneous. Indeed, one would expect a rapidly terminated process to be of little survival value to a young animal that relies on proximity to a particular individual for survival. The process of becoming familiar with an individual necessarily takes time, during which the young animal becomes familiar with views from different angles and in different types of illumination, and takes account of any changes in colour or morphology that the target might exhibit. Prolonged exposure to an object can impart a remarkable ability to discriminate between it and another object that resembles it quite closely. For example, after 4 × 50 min of exposure to a lifelike model of an adult fowl, chicks can distinguish between this and a novel model conspecific having similar plumage (Johnson & Horn, 1987).

A chick’s preference for the familiar stimulus does not change linearly with duration of exposure. Brief exposure of chicks to a stimulus will elicit approach (Bateson, 1966), but shortly afterwards there is a preference for slight novelty (P. S. Jackson & Bateson, 1974): with further exposure, this preference for novelty disappears, and a strong and persistent preference for the familiar emerges (Bateson & Jaeckel, 1976; P. S. Jackson & Bateson, 1974). This phenomenon was modelled by Bateson (Bateson, 1973), based on the hypothesis that during the imprinting process, a preference for the familiar and a preference for slight novelty both increase with time but at different rates, such that slight novelty becomes the more powerful attractor some time after the start of the imprinting process. This leads to further learning of objects’ characteristics, but a preference for slight novelty also stimulates exploration of the environment, entailing comparison of learned and novel information. P. S. Jackson and Bateson (1974) found evidence that experience of an object results in a tendency to explore novel objects. Bateson’s model proposes that, with time, the efficacies of familiarity and slight novelty become more similar in eliciting approach until they become identical and drive social preferences equally (Bateson, 1973).

Whereas a preference for slight novelty would enable a chick to accumulate information about an individual with which it is developing a filial relationship, the question arises as to whether the variety of sensory experiences thus acquired establish separate representations in the brain or are in some way integrated to be perceived collectively as features of a single individual. Evidence for the latter possibility was found by Bateson (1964), who pre-exposed chicks to a particular pattern on the walls of their rearing pen and then rewarded them for approaching a novel stimulus in a discrimination task where the familiar pattern (to which the chicks were presumably imprinted) was the negative stimulus. The discrimination, rewarding withdrawal from the familiar pattern, was learned more rapidly by the chicks that were familiar with the pattern than by chicks with no experience of the pattern. It was suggested that perceptual learning of the wall pattern had occurred, facilitating discrimination between the two stimuli during the test. A similar interpretation was placed on the finding that pre-exposure to two visual patterns on the same screen impaired subsequent learning of discrimination between the same two patterns by monkeys and chicks (Bateson & Chantrey, 1972): discrimination was more difficult if the patterns had previously been presented together. It was subsequently found that rapidly alternating exposure of chicks to two visual stimuli impaired their learning to discriminate between the stimuli in a subsequent operant task, relative to chicks exposed to the two stimuli in blocks about 30 min apart (Chantrey, 1974). The results support the hypothesis that chicks exposed to two stimuli in rapid alternation classify them together and that the chicks exposed in separate blocks classify the two stimuli separately. The results of a series of experiments support this notion (Honey et al., 1993, 1994, 1995; Montuori & Honey, 2015). To test this hypothesis in a situation somewhat closer to natural circumstances, Honey and Bateson (1996) exposed chicks to two views of a model hen (a Burmese jungle fowl, *Gallus gallus spadiceus*) presented alternately at two different rates. The chicks subjected to the faster rate performed significantly slower on a simultaneous discrimination test in which approach to one of the two views received a gentle heat reward.

A preference for slight novelty also occurs in the context of sexual imprinting. Bateson (1978) found that young adult male Japanese quail preferred female conspecifics of the opposite sex that were slightly different in appearance from the males’ nestmates. It was suggested that a sexual preference for slight novelty could bias mating behaviour in favour of an optimal degree of outbreeding. Quail of both sexes were found to prefer their first cousins to individuals with whom they were reared (Bateson, 1982). Domestic chickens display a similar preference for individuals that are slightly different from those with which they were reared, and this preference does not occur if the IMM, a forebrain region that stores information about a visual imprinting stimulus (see below) is lesioned shortly after hatching with the effect of preventing filial imprinting (Bolhuis et al., 1989). Despite the enormous cultural overlay to which human behaviour is subject, there is evidence of a similar tendency in human sexual behaviour to prefer slight novelty in a sexual partner, a trait associated with increased reproductive success (Bateson, 1983).

## 3. The Preference for a Familiar Object as a Measure of Imprinting

Some measure of preference for a visual stimulus to which a chick has been exposed is usually taken as a behavioural measure of visual imprinting (Bateson, 1966; Bolhuis, 1991; Sluckin, 1972). This usually employs data from an experiment in which naïve chicks are exposed either to stimulus A or to stimulus B and are subsequently tested by being given a choice between A and B in a preference test. The chicks typically move towards, or selectively direct other filial behaviour towards, the familiar stimulus. An estimate of the strength of the filial attachment may be obtained with approach data in the form of a preference score (McCabe et al., 1981), defined as the percentage of total approach activity during the preference test that is directed towards the familiar stimulus. The preference score can thus have a value between 0 (approach only towards the novel stimulus) and 50 (equal approach or no approach to either stimulus) to 100 (approach only towards the familiar stimulus). A preference score around 100 indicates strong imprinting.

Notwithstanding the extended time over which details of a visual imprinting stimulus can be learned, chicks can learn to discriminate between an imprinting stimulus and another stimulus very rapidly if the imprinting stimulus is particularly potent and the two stimuli in a discrimination test are sufficiently disparate. For example, training on either an internally illuminated rotating red box or an internally illuminated rotating blue cylinder (Horn, 1998) for 15 min is sufficient to generate a significant preference for the training stimulus that lasts for at least two days (Suge & McCabe, 2004). In the same series of experiments, it was shown that the preference score two days after training had approximately the same mean value whether the chicks were trained for 15 min or one hour.

**Figure 1:**
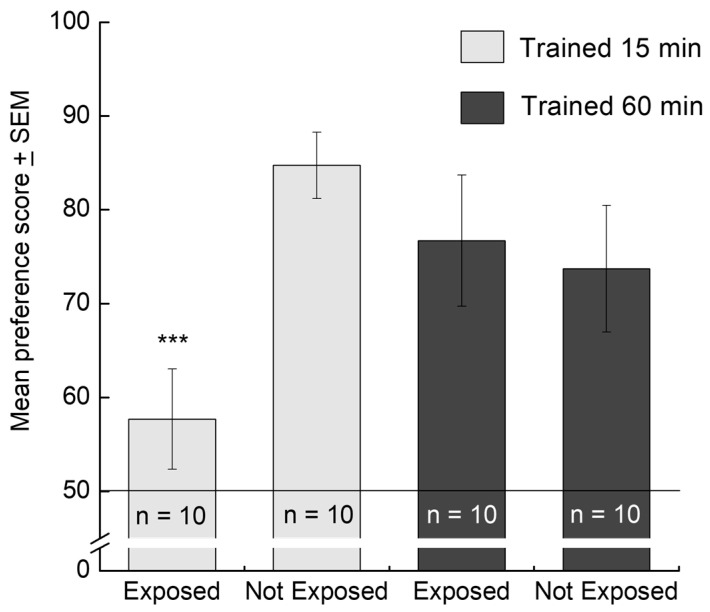
Preference scores of chicks trained on a visual imprinting stimuli for either 15 min or 1 h. Chicks were hatched and held in a dark incubator except when being trained and tested in a running wheel. Half of the chicks for each training time were trained on a rotating, internally illuminated red box and half on a rotating, internally illuminated blue cylinder while the maternal call of a hen was played in order to facilitate visual imprinting. On the following day, half of the chicks trained on each stimulus at each training time were exposed for 30 min to the stimulus that they had *not* been trained on (Exposed group), with no maternal call. The remaining chicks stayed in the dark incubator (Not Exposed). Each of the four groups in the figure were thus counterbalanced with respect to training stimulus. All chicks were then given a preference test in the absence of the maternal call, and the resulting preference scores were analysed with an analysis of variance. There was a significant interaction between Training Time (15 min, 1 h) and Novel Exposure Condition (Exposed, Not Exposed) (F[1,31] = 6.47, *p* = 0.016). That is, the effect of exposure to the novel stimulus depended on the time for which chicks were originally trained. The interaction is attributable to a significant reduction in preference score by exposure to a novel stimulus in the 15 min group (*** t[31] = 3.24, *p* = 0.003) but not the 60 min group. The two groups trained for 60 min, and the group trained for 15 min and not exposed to novelty were statistically homogeneous (from Katugampola et al. (2004)).

This result raises the question of whether, with these stimuli and training conditions, exposure for 15 min and for 1 h had equal effects on the chicks or whether there were effects of the longer period of exposure that were not apparent in the preference score. Figure 1 shows the result of an experiment to answer this question. Chicks were trained with either a red box or a blue cylinder, either for 15 min or 60 min. The recorded maternal call of a hen was played to the chicks during training—a way of increasing the effectiveness of visual imprinting (Smith & Bird, 1963). The next day, no maternal call was played, and half of the chicks in each subgroup received a second exposure to the stimulus that was *novel* to it. All chicks were then given a preference test with no maternal call, that is, requiring a preference to be expressed on the basis of only visual information. The results of this experiment are shown in Figure 1. Training for either 15 min or 60 min on Day 1 caused the chicks to be imprinted to their training stimuli, and the resulting preference persisted at similar levels on Day 2, provided that there was no exposure to novelty. However, if the chicks were exposed to the novel stimulus without sound on Day 2, the preference for the original (training) imprinting stimulus was significantly reduced. These results confirm that imprinting can proceed rapidly over the first 15 min of training and also show that subsequent training renders the preference score more resistant to the effect of an intercalated exposure to novelty. The neurobiological processes underlying this behaviour are discussed below.

## 4. Inference of Learning from Preference Score

Imprinting is expressed behaviourally as a preference for the stimulus with which a chick has been trained. Preference score, as defined above, is a convenient measure of that preference and hence of the extent to which a chick has learned about the training stimulus, provided that sources of variation such as that demonstrated in Figure 1 are controlled for. Different types of stimuli vary in their potency as imprinting stimuli (Bolhuis, 1991), and it aids interpretation of results if the two stimuli used in the preference test are approximately equipotent as imprinting stimuli. This can be shown by using each stimulus for training and ascertaining that there is no significant effect on preference score of stimulus type, as was the case for the experiment of Figure 1. The two training stimuli in that experiment were designed to be balanced in this respect.

It is possible to use just one imprinting stimulus for training and to use this and another stimulus, balanced as above, in a preference test to measure the extent of imprinting. An experimental design employing just one training stimulus and two balanced stimuli for testing has the merit of simplicity and is sometimes used in order to achieve a sufficiently large sample size where experimental resources are limited. A single training stimulus is also sufficient when naïve chicks’ preference for one of two stimuli is determined and then reversed by training with the less-preferred stimulus (Nicol & McCabe, 2014; Salzen & Meyer, 1968).

## 5. The Correlation Between a Neurobiological Measurement and Preference Score

The hypothesis that a neurobiological measure has a role in memory is supported if, as a result of training, the measure is significantly correlated with a behavioural index of memory such as preference score. The hypothesis is further supported where the correlation occurs in a brain region known to subserve memory (see below). If the chicks are trained with an imprinting stimulus for only a short period, they will typically show a range of preference scores between approximately 50 (no preference, no imprinting) and 100 (strong preference, strong imprinting). That is, some chicks, despite having been trained, will show no evidence of imprinting whereas others, for whatever reason, become imprinted more strongly. Interpolation of the corresponding regression line to a preference score of 100 gives the best estimate of the measure’s value when learning is robust and interpolation to preference score 50 estimates the measure’s value when no learning has occurred. It is also informative to investigate the measure in chicks from the same experimental cohort which have *not* been trained. These chicks do not learn anything about the training stimulus and have remained in the dark incubator without being subject to the handling, sensory stimulation, motor activity, emotional effects and other concomitants of the training procedure that are unrelated to learning. The effects (if any) of these processes extraneous to learning will be manifest as the difference between the mean value of untrained chicks and the value at preference score 50. If there is no significant difference between these two quantities, there is no evidence that side-effects of the training procedure affect the neurobiological measure. Any significant difference between the value at preference score 50 and the mean of untrained chicks estimates an effect of the training procedure unrelated to learning. One may also ascertain whether the value at preference score 100 is significantly different from the mean untrained value (or the value at preference score 50 if different from the untrained value). That difference, if statistically significant, shows that a significant change in the neurobiological measure can be achieved at the maximum preference score attainable. This type of analysis is illustrated in e.g., McCabe and Horn (1994), in which expression of the neural activity marker protein Fos in the domestic chick forebrain is plotted against preference score (see also Section 7 below).

When a neurobiological measure is intrinsic to learning that occurs during training, one might expect (i) a correlation with preference score characterised by its corresponding regression line and (ii) a residual variance about the regression line that is no lower (within sampling error) than the residual variance about the mean in untrained chicks. Put formally,

$$\sigma_{trained}^{2}=\sigma_{trained}^{2}.\rho^{2}+\sigma_{residual}^{2}$$

where

$\sigma_{trained}^{2}$ = variance of trained chicks;
ρ = correlation coefficient and $\rho^{2}$ is the proportion of variance attributable to the correlation;
$\sigma_{residual}^{2}$ = residual variance of trained chicks about the regression line.
Each component of the above model can be estimated experimentally.

If training *gives rise to* an association between the neurobiological measure and preference score, a correlation ρ > 0 adds to the variance of the trained chicks, and the residual variance about the regression line $\sigma_{residual}^{2}\;$ is the same as the population variance of the untrained chicks. This is because the trained and untrained chicks are sampled at random from the same population. In any one experiment, the sampled residual variance about the regression line and the sampled variance of untrained chicks would be expected to be statistically homogeneous. The sampled variance of trained chicks may or may not be *significantly* greater than the sampled variance of the untrained chicks, depending on sample size.

**Figure 2:**
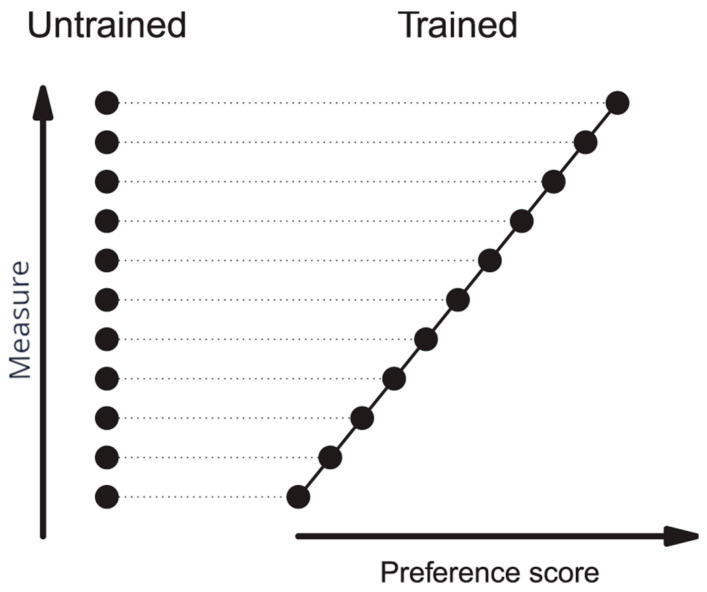
Theoretical illustration of how a physiological measure that is unaffected by training but associated with a predisposition to learn well can give rise to (i) a correlation with preference score after training and (ii) a residual variance about the regression line that is lower than the variance in the untrained condition. Measure denotes an arbitrary scale for the physiological measure; Untrained, hypothetical values of the measure in untrained chicks; and Trained, the same values after training and plotted against preference score. In this extreme example, there is a perfect correlation as a result of the measure’s association with a predisposition to learn well. The regression line passes through all the points, and all the variance in the untrained state is accounted for by the perfect correlation with preference score: the residual variance about the regression line is consequently zero. A residual variance about the regression line that is significantly lower than the variance in the untrained condition is evidence of a predisposition to learn well. Modified from Margvelani et al. (2018a).

If there is no effect of training on the neurobiological measure, there may be a correlation, but in that case the model predicts that $\sigma_{residual}^{2}$ is correspondingly reduced. To take an extreme example (Figure 2), suppose a neurobiological measure imparts a predisposition for efficient learning independent of training and is unaffected by training itself. Good potential learners attain high preference scores after training, and poor potential learners perform correspondingly poorly. There would then be a correlation with preference score simply because chicks are sorted according to their ability to learn, without training affecting the neurobiological measure at all. The variance attributable to the correlation is matched by a reduction in the residual variance around the regression line. In the illustrative theoretical example of Figure 2, there is a perfect correlation with preference score, and the residual variance about the regression in trained animals has decreased to zero: all the variation present in the untrained condition is accounted for by the correlation. Further discussion of this analysis is given by Margvelani et al. (2018a).

In practical terms, a significant reduction in residual variance about the regression line relative to the variance in untrained chicks indicates a predisposition to learn well rather than a learning-related change caused by training.

## 6. The IMM (Previously Named IMHV)

Compelling evidence indicates that the intermediate and medial mesopallium (IMM), a restricted part of the chick forebrain, is a site at which information about a visual imprinting stimulus is stored. The IMM was previously known as the intermediate and medial hyperstriatum ventrale (IMHV) until a new anatomical nomenclature for avian brain was introduced (Reiner et al., 2004).

The extensive evidence implicating the IMM in memory for an imprinting stimulus is well documented (Horn, 2004, 1985). The role of this region in imprinting was discovered by autoradiographic measurement of net RNA synthesis (the incorporation of radiolabelled uracil into macromolecules) in the chick forebrain during imprinting (Horn et al., 1979). Net RNA synthesis in the IMM was elevated depending on the amount the chicks learned during imprinting. The elevation could not be attributed to memory retention or recall, sensory exposure, motor activity, emotional changes or motivation. Encoding of information about the imprinting stimulus was the only plausible remaining explanation of these results, and this conclusion has been supported by subsequent research. Further experiments have shown both sides of the IMM to be involved in visual imprinting (Cipolla-Neto et al., 1982; Horn et al., 1983) and that functional N-methyl-D-aspartate glutamate receptors in the IMM are necessary for imprinting to occur (McCabe et al., 1992). Bilateral removal of the IMM before training prevents imprinting (McCabe et al., 1981). No behavioural side-effects were detected in any of the experiments in which the IMM was ablated or subject to pharmacological blockade.

**Figure 3:**
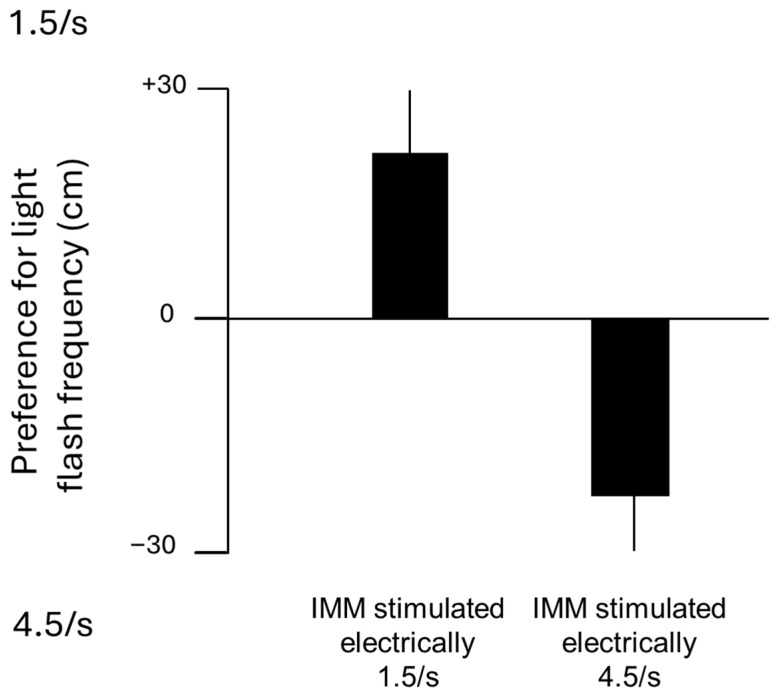
Generation of a preference for a frequency in the visual modality by previous electrical stimulation at that frequency in the IMM. The graph shows the mean preferences ± SEM of two groups of chicks, one having received electrical stimulation in the IMM at a frequency of 1.5 pulse trains s^−1^ and the other receiving electrical stimulation at 4.5 pulse trains s^−1^. To measure preference, each chick was placed in the middle of a two-metre-long railway. At one end of the railway was a silent visual stimulus flashing at 1.5 s^−1^, and at the other end was a silent, otherwise identical visual stimulus flashing at 4.5 s^−1^. Preference was expressed as the greater total distance in cm moved towards one or another stimulus during a 10 min test period. A positive distance arbitrarily denotes approach towards 1.5 s^−1^, and a negative maximum distance denotes approach towards 4.5 s^−1^. The testing apparatus is described fully by Bateson and Wainwright (1972). Electrodes were implanted bilaterally in the IMM under general anaesthesia. After recovery, chicks received electrical stimulation in the IMM at one of the two rates for a total of 5 h. They were then tested for their preferred frequency. Modified from McCabe et al. (1979).

Further evidence that the IMM can affect storage of information was provided by stimulating the IMM with trains of electrical pulses at one of two frequencies (1.5 s^−1^ or 4.5 s^−1^) for a total of five hours. When each chick was subsequently shown visual stimuli flashing at either of these two frequencies, the chicks selectively approached the visual stimulus flashing at the frequency of the electrical stimulation they had received (Figure 3). This effect did not occur when the stimulation was applied to two visual projection areas (entopallium and hyperpallium apicale) in the forebrain (McCabe et al., 1979). This result suggests that the IMM is capable of encoding temporal information.

Lesion experiments implicate the IMM in retention of the memory trace and indicate that different memory processes occur in the left and right sides of the IMM: If the right IMM is lesioned shortly after imprinting training, leaving only the left side of the IMM intact, that remaining side of the IMM is critical 24 h later for recognition of the imprinting stimulus. If, however, the order of unilateral ablation is reversed and the left IMM is lesioned first, the remaining right side enables information about the imprinting stimulus to appear outside the IMM and sustain retention in the absence of the IMM (Cipolla-Neto et al., 1982). There is therefore evidence of a role of the left IMM in retention over the 24 h after training and for the right IMM being capable of engaging further regions. Both sides of the IMM undergo learning-related changes over at least two days after the start of imprinting training (see below). With the right IMM intact, retention of memory for the imprinting stimulus ceases to become dependent on the IMM over the day following training. The location of the region outside the IMM (termed S’ by Cipolla-Neto et al. (1982)) that becomes capable of supporting retention is unknown, although a number of suggestions have been made (Behroozi et al., 2022; Solomonia et al., 2000; Tiunova et al., 2019). In an experiment in which chicks were prepared, by means of IMM lesions, to be either with or without S’ after training, the left IMM was found to support memory of the imprinting stimulus (cf. Cipolla-Neto et al., 1982), whereas classification together of visual stimuli by perceptual learning (see above) was evidently dependent on S’ (Honey et al., 1995). Both the IMM and S’ are necessary, in different ways, for memory of the imprinting stimulus one day after training.

## 7. Memory Processing in the IMM

**Figure 4:**
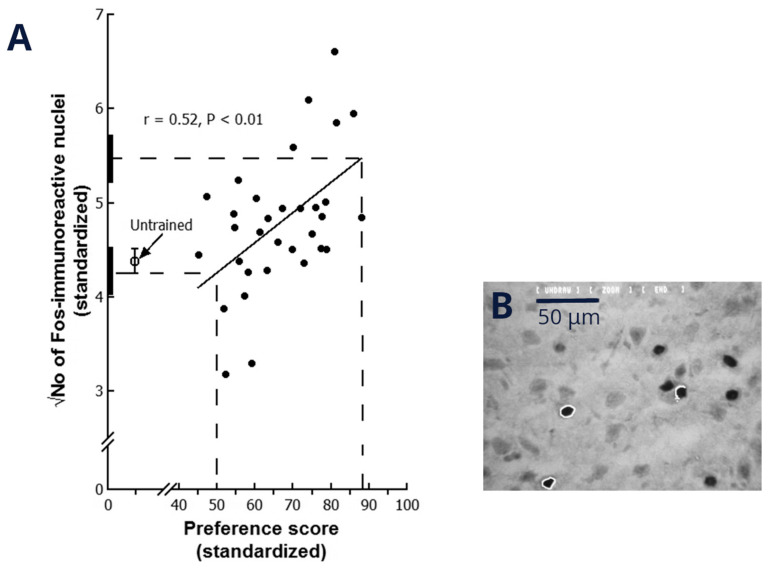
(**A**) Number of Fos-positive nuclei in the IMM plotted against preference score after chicks had been trained with a visual imprinting stimulus for 1 h, given a preference test 10 min after training and killed 50–70 min after training. Brains were removed, frozen, sectioned and processed for Fos immunocytochemistry. Each point is the mean number of Fos-positive nuclei in a 0.3 mm × 0.9 mm sampling frame; Fos counts were square-root-transformed for statistical analysis, and values on both axes were standardised to control for variation between replications of the experiment. Mean and SEM for untrained chicks are shown as an open circle and bar. Vertical dashed lines are drawn at preference score 50 (no evidence of imprinting) and 100 (strong imprinting). Horizontal dashed lines show the intercepts on the *y*-axis at these preference scores. Thick vertical bars on the *y*-axis denote the standard errors of these intercepts. In this example, from McCabe and Horn (1994), the mean for untrained chicks is not significantly different from the intercept at preference score 50, and the variance of trained chicks is significantly greater than the variance of the untrained chicks. The variance about the regression line is greater than the variance in untrained chicks, but not significantly so. The intercept at preference score 100 is significantly greater than the mean of untrained chicks, indicating a significant elevation in Fos expression at the maximum possible preference score. (**B**) Micrograph of section showing Fos-stained nuclei in the process of being selected automatically for counting according to shape, size and contrast with background. The light rings show nuclei being selected.

The expression of the activity marker protein Fos in neuronal nuclei in the IMM is elevated in a learning-related manner as a result of imprinting training for 1 h (McCabe & Horn, 1994); the numerical density of Fos-positive nuclei is correlated with preference score, and there is no evidence of side-effects of the training procedure (Figure 4). In this experiment, the variance of trained chicks was significantly greater than that of untrained chicks, and the residual variance about the regression line was not significantly lower than the variance of untrained chicks; the evidence thus indicates that the change in Fos expression arose with learning that resulted from training with the imprinting stimulus (see above). The effect is restricted to the subset of assumed inhibitory neurons, that is, immunopositive for γ-aminobutyric acid (GABA) and parvalbumin; cells double-stained for GABA and calbindin showed no change in Fos expression (Ambalavanar et al., 1999). Virtually all Fos expression in the IMM occurs in cells immunopositive for protein kinase C–γ (Ambalavanar et al., 1993). It has thus been possible to identify a subset of presumed inhibitory neurons in the IMM that are selectively activated during the first hour of memory formation. Further evidence implicating GABA receptors in imprinting comes from pharmacological experiments. Injection of GABA-B receptor antagonists or GABA-A agonists into the IMM on day 1 post-hatch were found to suppress imprinting; injection of GABA-B agonists or GABA-A antagonists on day 4 extended the sensitive period for imprinting (Aoki et al., 2018). Notwithstanding the implication of inhibitory neurons in the IMM shortly after training, there is also evidence at the same time of increased synaptic efficacy in glutamatergic (i.e., excitatory) neurons through enhanced phosphorylation and mobilisation of GluR1 neurotransmitter receptors (Solomonia et al., 2013).

How early in memory formation does the increase in Fos expression occur? Exposure to an imprinting stimulus for just 15 min gives rise to a preference for the stimulus lasting at least two days. The corresponding increase in Fos expression peaks approximately 2 h after the start of training, and the time course of the rise in Fos expression is similar when the training time is increased to one hour, the training time giving the correlation shown in Figure 4 (McCabe & Horn, 1994; Suge & McCabe, 2004). It is therefore likely that the increase in Fos expression reflects a very early stage of memory formation. This was confirmed by Suge et al. (2010), employing in-situ hybridisation to show that expression of the *c-fos* gene encoding Fos is at a maximum immediately after the end of a 15 min training period and falls to a much lower value 15 min later.

Further exposure to a visual stimulus over an extended period enables recognition of individual conspecifics (Johnson & Horn, 1987): training for a total of 4 × 50 min with the stuffed skin of a particular individual jungle fowl is sufficient to generate a significant preference for that individual over the stuffed skin of a novel individual. The ability to recognise the familiar individual was abolished by bilateral ablation of the IMM before training. Complementing this observation, neuronal responses in the IMM to individual companion chicks were found to be biased by previous exposure to these individuals for 9 h (Town, 2011a).

## 8. Electrophysiological Studies of the IMM

The IMM contains neurons that respond to visual stimuli, and their responsiveness to a visual imprinting stimulus changes during and after exposure to the stimulus. This effect was demonstrated in chicks trained with one of two imprinting stimuli (a rotating, internally illuminated red box or a rotating, internally illuminated blue cylinder) for a total of 100 min. Microelectrode recordings from the left IMM (Brown & Horn, 1994) and the right IMM (Nicol et al., 1995) the day after training revealed that the proportion of recording sites in the IMM showing significant responsiveness to the training stimulus was significantly higher than in untrained chicks, whichever of the two visual stimuli was used for training. There was no such effect on responsiveness to the alternative stimulus.

There is evidence for hemispheric asymmetry in the IMM with respect to neuronal responsiveness to a familiar imprinting stimulus (Moorman & Nicol, 2015; Nicol et al., 1995). Town (2011a) found that neurons in the right IMM showed a greater disparity of response than the left IMM to familiar and unfamiliar individuals previously encountered through social rearing for 9 h: unfamiliar individuals evoked a greater response than familiar individuals. In addition, social rearing after imprinting to an artificial stimulus (either a red box or a blue cylinder) reduced the preference for a familiar imprinting stimulus and revealed a hemispheric asymmetry in neuronal responsiveness in the IMM. In the left IMM, social rearing decreased responsiveness to the imprinting stimulus; in the right IMM, social rearing had the opposite effect (Town, 2011b).

**Figure 5:**
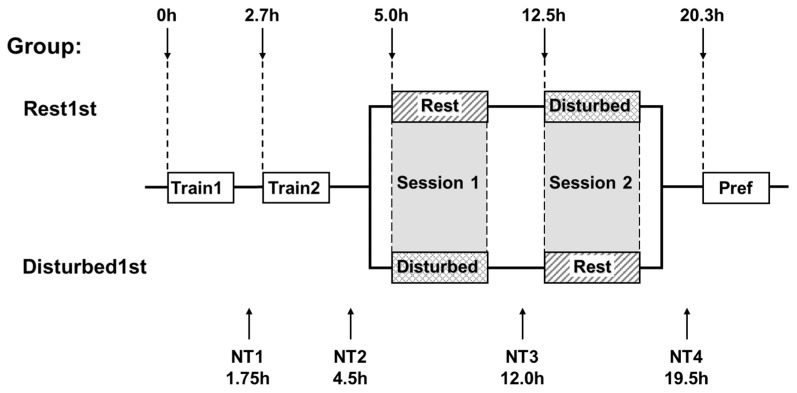
Experimental design of study by C. Jackson et al. (2008). Under general anaesthesia, a microelectrode assembly was fixed to the skulls of chicks ~10 h after hatching. The next day, after recovery from the anaesthesia, microelectrodes were introduced into the IMM, and recording of spike activity started, each chick placed in a running wheel. Chicks were trained by exposure to a rotating, internally illuminated red light (the imprinting stimulus, IS) for two 1 h periods, and after each training period neuronal responses to a number of visual stimuli studied (neuronal tests NT1 and NT2). The chicks were then subjected to either the Rest First or the Disturbed First experimental condition during Session 1. The Disturbed First chicks were subjected to the Disturbed treatment: slow movement of the running wheel for one minute at a random time in each successive 30 min period. The Rest First chicks were allowed to rest in darkness without disturbance and given the opportunity to sleep. After neuronal test NT3, each group of chicks received the alternative treatment, Disturbed or Rest, during Session 2. Neuronal responses were studied for the final time at test NT4, and each chick was then given a preference test in which the alternative stimulus (Avis) was a rotating, internally illuminated blue cylinder. The times of events are given in hours after the start of training (from C. Jackson et al. (2008)).

It is well established that ~24 h after the start of training, neuronal responsiveness in the IMM has become biassed towards an imprinting stimulus (IS) used for training. Tracking single IMM neurons across two one-hour training periods revealed considerable variability in responsiveness across time: the number of IS-responsive neurons in the IMM first rose, then fell, then rose again to approximately three times the pre-training level ~24 h after the start of training (Horn, 2004; Horn et al., 2001). The authors hypothesised from these results that individual IMM neurons can show such behaviour. This hypothesis was supported for a number of IMM neurons tracked for approximately 20 h from the start of imprinting training (C. Jackson et al., 2008). In this experiment, chicks were anaesthetised and prepared for microelectrode recording in the left and right IMM ~10 h after hatching. After recovery, ≥18 h post-hatch, microelectrodes were introduced into the IMM, and neuronal spike activity was recorded. The chicks were trained by being exposed for two 1 h training periods to an imprinting stimulus (IS—a rotating, internally illuminated red box) separated by one hour, during which responsiveness of IMM neurons to the IS and to an alternative visual stimulus (Avis) was measured. The Avis was a rotating, internally illuminated blue cylinder. Both of these stimuli were closely similar to the two stimuli used in previous experiments (Brown & Horn, 1994; Horn et al., 2001; Nicol et al., 1995). After a further test of neuronal responsiveness, the chicks were divided into two groups: The first was a Rest First group that was allowed to rest in darkness, in conditions conducive to sleep, from 5 h to 11 h after the start of training (Session 1). The remaining chicks were designated the Disturbed First Group and were subjected, during Session 1, to a one-minute revolution of the running wheel in which they were standing at a random time in each successive 30 min period. This procedure was designed to prevent sustained sleep. After a further test of neuronal responsiveness, the two groups received the Rest or Disturbed treatment, whichever they had not previously experienced, for a further 6 h (Session 2). After a final test of neuronal responsiveness, the chicks were given a preference test using the IS and Avis (Figure 5).

**Figure 6:**
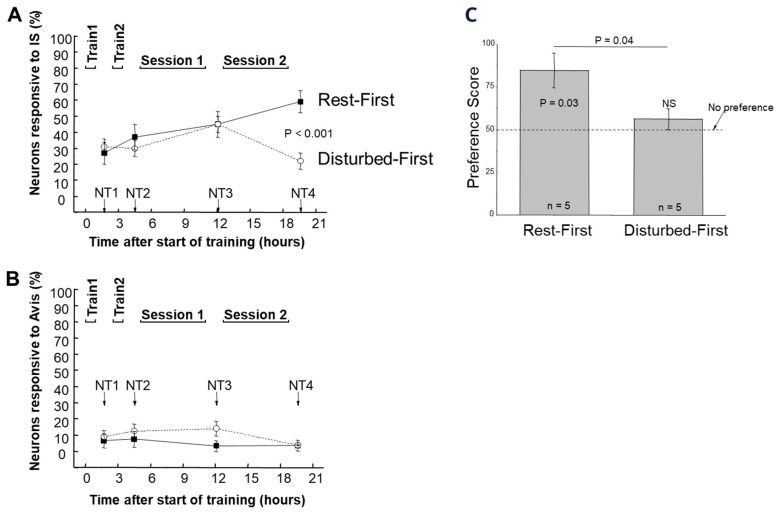
Results from C. Jackson et al. (2008). (**A**) Mean number of neurons significantly responsive to the imprinting stimulus (IS) throughout the experiment ± SEM. There was a significant difference between the two experimental groups, Rest First (filled squares) and Disturbed First (open circles), at neuronal test NT4, 19–20 h after the start of training. In the Rest First group there were significantly more responsive neurons at NT4 than at NT1. In the Disturbed First group the mean number of responsive neurons at NT4 was not significantly different from the mean number at NT1. (**B**) Mean number of neurons significantly responsive to the alternative visual stimulus (Avis), a rotating, internally illuminated blue cylinder. There was no significant change in this quantity in either group throughout the experiment. (**C**) Preference scores of the Rest First and Disturbed First groups ~20 h after the start of training, with Avis used as the alternative stimulus to IS, with which the chicks were trained. The Rest First group had a mean preference score that was significantly greater than the ‘no-preference’ level of 50 and significantly greater than the mean preference score of the Disturbed First group, which did not differ significantly from 50.

The Rest First group became imprinted to the IS, having a mean preference score of 84.8 ± 10.1 SEM at the end of the experiment. In contrast, the Disturbed First group showed no evidence of imprinting, with a significantly lower mean preference score of 56.2 ± 6.20. There was also a marked difference in neuronal responsiveness in the two groups (Figure 6). There was a highly significant difference in neuronal responsiveness to the IS between the Rest First and Disturbed First groups at the final test. The Rest First group had approximately doubled its responsiveness to the IS relative to that at the first neuronal test. However, the Disturbed First group’s neuronal responsiveness to the IS had declined to approximately that of the first test. Responsiveness to the Avis scarcely changed at all throughout the experiment (Figure 6).

The effective early sleep period (but not the ineffective late sleep period) was characterised by a significantly increased proportion of electroencephalographic energy in the low theta frequency band (5–6 Hz), counterbalanced by a reduction in proportion of energy between 9 Hz and 24 Hz (C. Jackson et al., 2008).

The increased neuronal responsiveness to the IS at the end of the experiment was attributable to sleep during Session 1 and not to sleep disruption during Session 2 because the increased responsiveness occurred in the experiment by Horn et al. (2001), when there was no experimental disruption of sleep.

The unstable, sleep-dependent behaviour of neuronal IS responsiveness in the IMM suggests a period of consolidation over the day following training, which may be related to some or all of the learning-related changes in the IMM observed over the hours following training (Solomonia & McCabe, 2015). It was pointed out (Horn, 2004; Nicol et al., 2003) that the instability of responsiveness after training is not consistent with a simple so-called Hebbian assembly (Hebb, 1949), which is often proposed as a mechanism for memory, whereby a group of interconnected neurons undergo activity-dependent strengthening of their connections, implying a monotonic increase in responsiveness as learning and consolidation progress. A further finding inconsistent with a Hebbian cell assembly (Horn, 2004) is that no significant change was found in the level of cross-correlation between spikes from neurons recorded by the same electrode pair in the IMM (Nicol et al., 2003). One would expect an increase in correlation of spike timing if a Hebbian cell assembly developed as a result of training.

The effect of sleep on memory for an imprinting stimulus was investigated further by Nicol and McCabe (2014). As a precaution against the preference for one or another imprinting stimulus being unbalanced, chicks were hatched and reared in darkness until ~12 h post-hatch, when they were given a preference test (P0) before training, by exposure to the two imprinting stimuli later used for training—red box (RB) and blue cylinder (BC). The chicks were then anaesthetised, and stainless-steel stimulating electrodes were implanted in the left and right frontal bones of the skull. The next day, after recovery from the anaesthetic, the chicks were exposed for two one-hour sessions (separated by 90 min in a dark incubator) to the imprinting stimulus that was less preferred during test P0. Imprinting would therefore be inferred if there were a reversal of a chick’s naïve preferences. The chicks then received a second preference test (P1), followed by 6 h of sleep disturbance in darkness by turning the chicks’ running wheel (cf. C. Jackson et al., 2008). During the first three hours of disturbance, the chicks were allocated to one of four experimental conditions: sinusoidal electrical stimulation through the implanted electrodes (2 µA peak to peak) at either 0.75 Hz or theta frequency (6 Hz), sham-implanted but no electrical stimulation and unoperated controls. The chicks received two further preference tests at approximately 8 h (PT2) and 16 h (PT3) after the end of the period of disturbance. The mean preference score for all chicks at PT1 (73.0 ± 2.0 SEM) was significantly higher than that at P0 (39.8 ± 2.0), showing that imprinting had occurred. The mean preference scores of the four experimental groups did not differ significantly from one another in these first two tests. After the period of sleep disruption, the combined mean preference score of sham-implanted and unoperated controls at PT2 and PT3 (64.1 ± 3.0) was significantly reduced relative to PT1 as expected from C. Jackson et al. (2008). In contrast, the combined mean preference score for PT2 and PT3 for both electrically stimulated groups of chicks (73.2 ± 3.1) was almost identical to the combined score at PT1. Electrical stimulation therefore prevented the delayed fall in preference score following sleep disturbance, with no significant difference between the effects of slow (0.75 Hz) and theta-frequency (6 Hz) stimulation.

This result is reminiscent of experiments in which transcranial electrical stimulation at slow-wave sleep frequency can improve recall in human subjects (reviewed in Lutz et al., 2026). This result indicates that in so far as the effects of transcranial electrical stimulation on memory are similar in chicks and humans, the chick may offer a fruitful means of experimentally investigating the neuronal processes underlying memory consolidation during sleep.

## 9. Learning-Related Synaptic Changes in the IMM Following Imprinting Training

Electrophysiological responsiveness in the IMM to the imprinting stimulus is increased approximately 2–4 h after the start of training, returns to near baseline by ~8 h, then increases again to 2–3 times the baseline level by ~24 h after the start of training (Horn et al., 2001; C. Jackson et al., 2008). These changes are accompanied by learning-related structural and functional changes in synapses in the IMM and, in the later stages, changes in protein molecules implicated in synaptic plasticity. The mean profile length of the apposition zone of axospinous (putative excitatory) synapses in the left IMM is increased ~6.5 h after the start of training (Bradley et al., 1981; Horn et al., 1985), and the numerical density of NMDA receptors in the left IMM is increased in a learning-related manner 11 h after the start of training (McCabe & Horn, 1991, 1988). The estimated increase in apposition area is consistent with NMDA receptors being added to the extra sub-synaptic membrane at the same areal density as before training (McCabe & Horn, 1988). In addition to the increase in presumed inhibitory activity in the first hour of training (Ambalavanar et al., 1999; Suge et al., 2010; Suge & McCabe, 2004), there is an increase in phosphorylation of the excitatory GluA1 glutamate receptor, at its serine-831 site, approximately 2 h after the start of a one-hour training period (Solomonia et al., 2013). This phosphorylation is probably due to an increase in the rate at which the receptors are inserted into the postsynaptic membrane and to increased channel conductance through an increased channel opening rate (Derkach et al., 1999; Hu et al., 2007; Kristensen et al., 2011). These effects on the GluA1 receptor, together with modifications of neurotransmitter release, are affected by calcium/calmodulin-dependent protein kinase II (CaMKII), activated by exposure to calcium/calmodulin and autophosphorylation of threonine-286 in its α-subunit. The level of threonine-286 phosphorylation of α-CaMKII in the IMM is also raised as a result of learning 2 h after the start of training (Solomonia et al., 2005).

Consistent with the learning-related synaptic modifications in the IMM mentioned above, imprinting training leads to learning-related changes in calcium-dependent potassium-stimulated release of neurotransmitters in the IMM—release that is assumed to be the amount of available physiologically releasable neuroptransmitter from synapses. Release of GABA and taurine was enhanced in a learning-related manner 4.5 and 11 h after the start of imprinting training (McCabe et al., 2001; Meredith et al., 2004). The inhibitory role of GABA is well established; a number of functions have been proposed for taurine in the central nervous system, including that of neurotransmitter and neuromodulator (Wu & Prentice, 2010). By ~24 h after the start of training, the release of taurine and GABA was no longer correlated with preference score. In contrast, calcium-dependent potassium-stimulated release of glutamate was elevated relative to the level in control, untrained chicks (Meredith et al., 2004).

There is thus abundant evidence of learning-related modifications of excitatory, inhibitory and possibly neuromodulatory synaptic transmission in the left IMM in the hours following imprinting training. By 24 h after the start of training, learning-related changes in many molecules in the IMM have been detected, all predominating in, or restricted to, the left IMM. Many of these changes are genomic or proteomic; some are a result of learning, and others reflect a predisposition to learn. The learning-related changes occurring ~24 h after training are summarised in Table 1. A comprehensive transcriptomic study of the IMM currently available in preprint form (Lagani et al., 2025) has implicated long non-coding RNA both in imprinting and in a predisposition to learn well.

## 10. Implications for Human Development: Predispositions

Human neonates learn rapidly, recognize stimuli that they have previously encountered, and show some remarkable similarities to imprinting and related processes studied in precocial birds. Non-invasive techniques, such as the recording of event-related potentials, can give insights into the time course of recognition during the first year of human life (Rigato et al., 2025), which may be compared with the more detailed electrophysiological work in chicks (Horn et al., 2001; C. Jackson et al., 2008). One should not of course be naïve in making comparisons across large evolutionary distances and profound differences in cognitive capacity and complexity. Nevertheless, there are striking similarities. As regards cognitive capacity and complexity, there is evidence that ducklings are capable of relational concept learning, an ability present in human infants at 7–9 months of age (Ferry et al., 2015). Ducklings, imprinted to a pair of objects of the same or different shape, preferred the familiar configuration (i.e., same or different) when given a choice between two pairs of novel objects, one pair with the same shape and the other pair with different shapes. A similar result was obtained when colour rather than shape was manipulated (Martinho & Kacelnik, 2016).

The importance of sleep for memory in human subjects is well established (Diekelmann & Born, 2010; Lutz et al., 2026). Sleep is also important for retention of chicks’ memory for a visual imprinting stimulus (C. Jackson et al., 2008). In studying the time course of neuronal responsiveness to the imprinting stimulus, it was found that sleep had to occur during a particular period after training for neuronal responsiveness and memory to be preserved, revealing a post-training period of neuronal instability and sensitivity to sleep. Memory can be maintained by electrical stimulation of the brain at theta (6 Hz) or slow (0.75 Hz) frequencies without phase-locking to spontaneous electroencephalographic activity (Nicol & McCabe, 2014). Imprinting thus provides an opportunity to investigate mechanistic interaction between sleep and memory that is experimentally tractable and which may contribute to the understanding of the neurobiology of sleep in human subjects.

Young animals are attracted to some visual stimuli more readily than others without having previously been exposed to these stimuli. Newly hatched domestic chicks show a predilection for a model of a jungle fowl (Horn & McCabe, 1984). This predisposition is released by mild, non-specific sensory stimulation shortly after hatching: if the chicks are allowed to sit quietly in a dark incubator, the predisposition does not appear, but the mild stress of handling the chicks—taking them from a dark incubator and putting them into a running wheel—is enough to trigger the predisposition. It seems likely that mild stress such as that which would be caused by isolation would give rise to the predisposition and bias a chick’s approach towards conspecifics, since chickens spend much of their time in social groups. The predisposition need not be so precise as to attract only to a specific individual—that could be maladaptive if the parent were absent—and indeed there may be a limit to the specification that is possible in a developmental program acting in the absence of visual input (Bolhuis et al., 1985). The predisposition was found to be targeted on the features of the head and neck (Johnson & Horn, 1988), particularly facial features (Rosa-Salva et al., 2019, 2009), and to be sensitive to the level of plasma androgen (Bolhuis et al., 1986). It is not, however, affected by injection of the anti-adrenergic neurotoxin DSP4 (Davies et al., 1992) at a level that impairs imprinting (Davies et al., 1985) and is therefore mediated by a neural mechanism different from the one responsible for imprinting. The predisposition has a sensitive period (Johnson et al., 1989) and is not species-specific: chicks with the predisposition will also approach a stuffed skin of a duck or even of a predator (a polecat) (Johnson et al., 1985). Chicks are now known to be predisposed to approach stimuli showing animacy and/or biological motion (movement of groups of neighbouring image elements in the same direction), tending to direct chicks’ attention towards living animals (Vallortigara, 2021; Vallortigara et al., 2005). The function of the predisposition has been suggested (Horn, 1985; Johnson et al., 1985) to direct chicks’ attention and cause them to approach animate objects, allowing for learning of the objects’ features through imprinting—the so-called two-process theory of filial imprinting.

The above observations in chicks led to the discovery of a similar predisposition for human neonates to attend to human facial patterns (Di Giorgio et al., 2017; Johnson et al., 1991). A two-process theory, based on chick imprinting research, was proposed for the development of face recognition in humans (Johnson et al., 1991; Morton & Johnson, 1991). In this theory, “Conspec” is a process analogous to chicks’ predisposition to attend to faces and “Conlern” a process corresponding to learning about a face once attention is engaged. A discussion of possible neural mechanisms underlying the two processes is given by Johnson et al. (2015). It is notable that lack of interest in features characterising living animals has been described in children with autistic spectrum disorder, suggesting that chicks may afford a way of understanding neural processes underlying this disorder (Klin et al., 2015; Matsushima et al., 2024).

Note that this predisposition is distinct from the predisposition to learn well that is associated with the activity of certain molecules in the IMM (cf. Figure 2); it is not known whether these predispositions are related.

## 11. Conclusions

I have reviewed studies of imprinting in the domestic chick that have advanced the understanding of a number of fields: neural mechanisms of memory and the effect of sleep, localisation of memory processing in the brain, the role of predispositions and learned behaviour in social bonding, perceptual learning and recognition memory in the establishment of filial relationships, the connection between filial and sexual imprinting, the contribution of early experience to optimal outbreeding and the identification of molecular species associated with early learning. Contemporary transcriptomic studies provide a molecular basis for identifying homologies in avian and mammalian brains. Whether the similarities between imprinting in precocial birds and social bonding in primates result from evolutionary convergence or conservation, these similarities testify to the versatility of the neuron, the properties of which have not changed fundamentally in hundreds of millions of years. These similarities suggest that there is a limit to the number of ways that nerve cells can control behaviour. If that is so, investigation of the properties of neuronal assemblies and gene expression in neurons and glial cells in an experimentally tractable species like the domestic chick are likely to be an efficient way of understanding mechanisms of affiliative behaviour held in common by different species. I have, accordingly, outlined some of the ways in which imprinting studies may aid the understanding of human neurological and social development.

## Figures and Tables

**Table 1:** Molecular species in the IMM showing a correlation with preference score ~24 h after training on an imprinting stimulus for 1 h. It is indicated whether the correlation was attributable either to learning during training or to a predisposition to learn well that was independent of training. Side of occurrence in the IMM is also given. Preference score was measured 10 min after the end of training.

Molecule	Ascribed to Learning During Training or to Predisposition to Learn Well	Side of IMM	Reference
Clathrin heavy chain protein	Learning	Left	Solomonia et al. (1997)
Neural cell adhesion molecule proteins	Learning	Left	Solomonia et al. (1998)
MARCKS protein, Amyloid precursor protein	Learning	Left	Solomonia et al. (2003)
Membrane-bound (unphosphorylated) MARCKS protein	Learning	Data from left and right IMM were pooled in this analysis	Solomonia et al. (2008)
Cytochrome c oxidase protein subunits I and II, α-fodrin protein	Learning	Left	Solomonia et al. (2011)
Membrane-bound cognin protein, Protein resembling P32 subunit of splicing factor SF2 (membrane fraction), Dynamin-1 protein, P38 protein in membrane-mitochondrial fraction, Voltage-dependent anionic channel 1 protein, heterogeneous nuclear ribonucleoprotein A2/B1, Mitochondrial transcription factor A protein, Nuclear respiratory factor 1 protein	Learning	Left	Meparishvili et al. (2015)
Mitofusin-1 protein, Dynamin-related protein-1	Learning	Left	Margvelani et al. (2018b)
Micro-RNA gga-miR-130b-3p, Membrane-associated cytoplasmic polyadenylation element binding protein 3 (CPEB-3)	Predisposition	Left	Margvelani et al. (2018a)
Mitochondrial ATP synthase (b5 subunit) protein, Na/K ATPase protein (α2 subunit)	Learning	Left	Chitadze et al. (2020)
Src tyrosine kinase protein, Tyrosine 527-phosphorylated Src (inhibited form)	Learning	Left	Meparishvili et al. (2021)
Tyrosine 416-phosphorylated Src (activated form) as % of total Src	Predisposition (negative correlation)	Left	“
CPEB-3 protein, aggregated form	Predisposition	Left	Chitadze et al. (2023)
Src-NADH2 complex	Learning (negative correlation)	Right	Chitadze et al. (2024)
Long non-coding RNA ENSGALG00010007489, protein LUC7L, protein Retinoid-related orphan receptor-513-α (RORA), Forkhead box protein P2 (FOXP2)	Learning	Left	Lagani et al. (2025)
Long-non-coding RNA ENSGALG00010026609, Roundabout guidance receptor 1 (ROBO-1)	Predisposition	Left	“

## Data Availability

No new data were created or analysed in this study.

## Abbreviations

The following abbreviations are used in this manuscript:

SEM	Standard error of the mean
RNA	Ribonucleic acid
